# Genome-wide recruitment profiling of transcription factor Crz1 in response to high pH stress

**DOI:** 10.1186/s12864-016-3006-6

**Published:** 2016-08-20

**Authors:** Alicia Roque, Silvia Petrezsélyová, Albert Serra-Cardona, Joaquín Ariño

**Affiliations:** 1 Departament de Bioquímica i Biologia Molecular, Universitat Autònoma de Barcelona, Cerdanyola del Vallès, 08193 Barcelona, Spain; 2 Institut de Biotecnologia i Biomedicina, Universitat Autònoma de Barcelona, Cerdanyola del Vallès, 08193 Barcelona, Spain; 3 Present Address: Transgenic Models of Diseases & Transgenic Unit, Institute of Molecular Genetics of the ASCR, v. v. i., BIOCEV, Průmyslová 595, Vestec, CZ-252 42 Czech Republic

**Keywords:** Alkaline pH stress, Calcineurin signaling, Crz1 recruitment, Consensus binding sequence, *Saccharomyces cerevisiae*

## Abstract

**Background:**

Exposure of the budding *Saccharomyces cerevisiae* to an alkaline environment produces a robust transcriptional response involving hundreds of genes. Part of this response is triggered by an almost immediate burst of calcium that activates the Ser/Thr protein phosphatase calcineurin. Activated calcineurin dephosphorylates the transcription factor (TF) Crz1, which moves to the nucleus and binds to calcineurin/Crz1 responsive gene promoters. In this work we present a genome-wide study of the binding of Crz1 to gene promoters in response to high pH stress.

**Results:**

Environmental alkalinization promoted a time-dependent recruitment of Crz1 to 152 intergenic regions, the vast majority between 1 and 5 min upon stress onset. Positional evaluation of the genomic coordinates combined with existing transcriptional studies allowed identifying 140 genes likely responsive to Crz1 regulation. Gene Ontology analysis confirmed the relevant impact of calcineurin/Crz1 on a set of genes involved in glucose utilization, and uncovered novel targets, such as genes responsible for trehalose metabolism. We also identified over a dozen of genes encoding TFs that are likely under the control of Crz1, suggesting a possible mechanism for amplification of the signal at the transcription level. Further analysis of the binding sites allowed refining the consensus sequence for Crz1 binding to gene promoters and the effect of chromatin accessibility in the timing of Crz1 recruitment to promoters.

**Conclusions:**

The present work defines at the genomic-wide level the kinetics of binding of Crz1 to gene promoters in response to alkaline stress, confirms diverse previously known Crz1 targets and identifies many putative novel ones. Because of the relevance of calcineurin/Crz1 in signaling diverse stress conditions, our data will contribute to understand the transcriptional response in other circumstances that also involve calcium signaling, such as exposition to sexual pheromones or saline stress.

**Electronic supplementary material:**

The online version of this article (doi:10.1186/s12864-016-3006-6) contains supplementary material, which is available to authorized users.

## Background

Development of proper responses to changes in the extracellular environment is crucial for cell survival. The budding yeast *Saccharomyces cerevisiae* vigorously proliferates in acidic media, but even a moderate alkalinization (pH ~8.0) of the medium is hardly tolerated by most yeast strains and, therefore, it represents a stressful situation. To confront alkaline pH stress, *S. cerevisiae* mounts a robust and transient transcriptional response affecting several hundred of genes [1–4]. This response is the result of the coordinate activation of diverse signaling pathways that, in some cases, have been characterized in detail (see [5, 6] for recent reviews).

Among the most relevant pathways determining the transcriptional response under high pH stress one is the triggered by an increase in cytosolic calcium levels and mediated by calcineurin, a Ser/Thr protein phosphatase regulated by calcium/calmodulin. In budding yeast calcium is a common second messenger for diverse stimuli, such as exposure to mating pheromones, high salt or osmolarity, endoplasmic reticulum stress, and others [see [7] and references therein]. It has been documented that high pH stress triggers an almost immediate, sharp and transient peak of cytosolic calcium that results in activation of calcineurin [4]. In budding yeast, calcineurin is a heterodimer, composed of one of two redundant catalytic subunits (encoded by *CNA1* and *CNA2*), plus a regulatory subunit, encoded by only one gene, *CNB1* [8]. Activation of calcineurin in response to rising cytosolic calcium levels impacts on several downstream targets, but a major one is the zinc-finger transcription factor (TF) Crz1/Tcn1/Hal8 (from now on, Crz1). Calcineurin dephosphorylates Crz1 [9–11], which then enters the nucleus and binds to specific sequences present in the promoters of calcineurin-responsive genes (e.g. *FKS2*), defined as 5′-TG(A/C)GCCNC-3' and known as *C*alcineurin *D*ependent *R*esponse *E*lement (CDRE). The participation of calcineurin in regulating the expression of the *ENA1* Na^+^-ATPase was reported long time ago [12–14]. Early work by Yoshimoto and coworkers showed that expression of a subset (around 160) of those genes induced upon addition of calcium or sodium cations to the medium was largely dependent on calcineurin and, with very few exceptions, also on the presence of Crz1 [15]. Subsequently, Viladevall and coworkers identified 27 genes whose induction by alkaline pH stress was dependent, at least in part, on the presence of calcineurin [4]. Such induction was in most cases also dependent on Crz1.

*In vitro* binding of Crz1 to specific CDRE sequences was demonstrated long time ago for several examples, such as *FKS2* or *ENA1* [9, 16], and the nature of the binding site subsequently explored by *in vitro* selection [15]. *In vivo* recruitment of Crz1 to the promoters of targets genes under specific stress conditions has been experimentally proved only in a very few cases [17, 18]. It must be stressed that although large-scale studies have been undertaken in the past to address the *in vivo* specificity of many yeast transcription factors [19] these approaches often fail to identify biologically significant binding events because the TF does not bind DNA under the assay conditions. Crz1 would be an example of this kind of TF, since under normal growth conditions is located in the cytoplasm. We present here a genome-wide analysis of the binding of Crz1 to promoter regions in response to alkalinization of the medium, a condition that is known to promote Crz1 activation and its nuclear localization. We show time-dependent recruitment of Crz1 to 152 intergenic regions. We also analyze the possible physiological relevance of such interactions, and use this information to refine the consensus sequence for Crz1 promoter recruitment.

## Methods

### ChIP-Sequencing (ChIP-Seq) assays

Construction of strain SP020, which expresses the Crz1 protein C-terminally tagged with the 3xHA epitope was previously described [18]. For chromatin immunoprecipitation (ChIP) experiments strain SP020 was grown overnight to mid-exponential phase at 28 °C in liquid rich medium (YPD; 10 g/l yeast extract, 20 g/l peptone and 20 g/l dextrose) and then resuspended in fresh YPD media containing 50 mM TAPS adjusted to pH 5.5 to OD_600_ = 0.2. Cells were grown for 4–5 h to reach OD_600_ = 0.6–0.8. At time 0, the pH of the culture was raised to 8.0 by adding 1 M KOH and growth resumed. Forty ml of cultures were taken at the appropriate times (1, 2, 5 and 20 min) and made 1 % formaldehyde to crosslink proteins and DNA. From this point the procedure was as described in [18, 20] with the following modifications: i) chromatin was fragmented using a Bioruptor Plus UCD-300 equipment (Diagenode) provided with a cooling system (4 °C) for 30 cycles (high intensity; 30 s of sonication followed by 60 s pause) to generate fragments of ≤ 500 bp length; ii) the supernatant was pre-cleared with protein G sepharose^TM^ fast flow (GE Healthcare, #17-0618-01) beads for 1 h at 4 °C and then the pre-cleared cell lysate was incubated with 1 μg of polyclonal anti-HA ChIP-grade (Abcam, #ab9110) antibody overnight at 4 °C; iii) anti-HA-Crz1-DNA complexes were collected with protein-G-sepharose beads incubating for 1 h at 4 °C, and iv) after reverse crosslinking, DNA was extracted using a NucleoSpin® Gel and PCR Clean-up kit (Macherey Nagel, #740609) and NTB buffer (Macherey-Nagel, #740595). ChIP libraries were prepared using the TruSeq ChIP Sample Preparation Kit (Illumina) and then subjected to paired-end deep sequencing with the MiSeq Reagent Kit v2 (300 cycles) to provide reads of around 150 nt.

### Data analysis

FASTQ files were mapped using Bowtie2 (version 2.1.0, “end-to-end” mapping and “very sensitive” setting) to generate the corresponding SAM files [21]. Mapped reads (1.9 to 3.7 millions/time point) were sorted and indexed using IGV tools [22]. Subsequent analysis was performed using the SeqMonk software (Babraham Institute, http://www.bioinformatics.babraham.ac.uk/projects/seqmonk/). The *S. cerevisiae* EF4 (sacCer3) genomic data was employed. The chromosomal coordinates of the intergenic regions and information on the flanking genes were obtained from the Saccharomyces Genome Database using the YeastMine tool. This information was loaded into SeqMonk as an Annotation set and was used to generate 6303 intergenic probes. Subsequently, each of these probes was tiled in contiguous sections (bins) of 50 nt. This generated a total number of 65198 probes that were quantitated by the read count quantitation method, with identical reads removed and correction for total read count referred to the largest Dataset. Peaks were detected using an in-house VBA-based algorithm that detected a minimum of two-fold enrichment over the genome average at a given time-point. The algorithm included a functional filter to select for profiles compatible with the kinetics of Crz1 entry to the nucleus previously observed for this same kind of stress (e.g., that the enrichment would correspond at times 2 and/or 5 min after stress) [17, 23]. Our selection was further confirmed with two standard peak-calling methods, Model-based Analysis for ChIP-Seq (MACS) [24] and Genome wide Event finding and Motif discovery (GEM) [25]. MACS uses a Poisson test to rank their candidate peaks and is considered one of the methods with higher sensitivity, while GEM uses Binomial test for statistical testing and stands out by the accuracy of the location of the peaks [26]. The optimal parameters for initial peak calling with MACS were as follows: bandwidth, 130 nt; minimum fold enrichment, 2; *p*-value cut-off, 10^−3^. GEM was run using the default read distribution, sacCer3 genome sizes available at the UCSC Genome Browser (http://genome.ucsc.edu/), and a minimum fold enrichment of two.

### Cluster analysis

Cluster analysis was performed with Cluster 3.0 and the data corresponding to the maximum fold-change defining the peak of the assigned genes at 0, 1, 2, 5 and 20 min. Results were visualized with Java Treeview 1.1.6 [27, 28].

### Gene Ontology analysis

Significant shared Gene Ontology (GO) terms by all the assigned genes or for specific groups were searched using the GO Term Finder (version 0.83) available at the Saccharomyces Genome Database with a *p*-value cutoff of 0.01.

### Putative CDRE search

To search for putative CDREs at the ChIP-Seq peaks a matrix scan of the *S. cerevisiae* genome was performed at the PWMTools website (http://ccg.vital-it.ch/pwmtools/pwmscan.php) of the Swiss Bioinformatics Institute. The position weight matrix (PWM) for Crz1 reported by Badis and coworkers [29] was used as a reference with a *p*-value cutoff of 10^−3^. In parallel, a pattern search of the intergenic sequences using the core sequence GCC was performed with the Pattern Matching tool from the Saccharomyces Genome Database. All the information was loaded into SeqMonk as an Annotation for further analysis. Transcription start sites (TSS) were obtained from Xu and coworkers [30].

### Position weight matrix refinement

The CDREs mapping at the center of each ChIP-Seq peak were selected and grouped based on the strength of the Crz1 recruitment. A new PWM was generated for each group using an in-house Java application. A sequence weblogo was created for each CDREs group at http://weblogo.berkeley.edu/logo.cgi.

### Chromatin structure analysis

Nucleosome positioning and core histone modifications present in the regions recruiting Crz1 were analyzed using data available at the Saccharomyces Genome Database. Specifically, the data reported by Mavrich et al. [31] were used as reference for nucleosome positioning and the data reported by Guillemette et al. [32] were used as reference for core histone modifications (H3K4me3 and H3K4ac).

## Results and discusion

### Identification of genomic regions recruiting Crz1 upon high pH stress

**Fig. 1:**
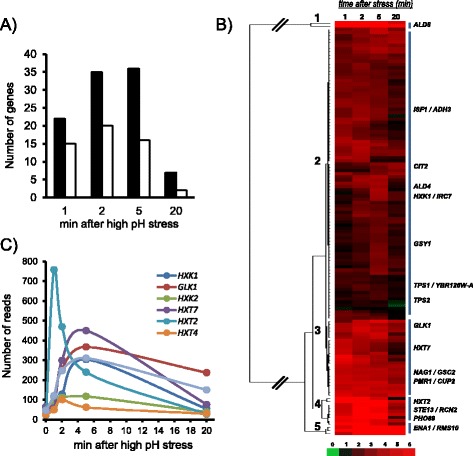
Kinetics of Crz1 recruitment to gene promoters upon high pH stress. **a** Time-course of accumulation of Crz1 at intergenic regions. The time-point at which the higher number of reads was accumulated at the peak region was identified for the 100 intergenic regions for which a given gene could be assigned (*filled bars*) and for the 52 divergent promoters (*empty bars*). **b** The ratio of the number of reads at the peak of each intergenic region at a given time and at time zero was calculated (fold-change) and the resulting values (expressed as log2) clustered using Cluster 3.0 (Euclidean distance, complete linkage) and represented with Java Treeview v1.1.6. The positions in the cluster of promoters corresponding to genes known to respond to calcineurin/Crz1 activation or for which evidence for *in vitro* or *in vivo* binding of Crz1 exists are represented at the right. **c** The time-course distribution of the number of reads at the Crz1 peak of recruitment is shown for diverse promoters of genes involved in glucose transport and phosphorylation

Our analysis of ChIP-Seq data at intergenic regions allowed identifying 152 regions with increased accumulation of reads (at least 2-fold over the genome average at one or more time-points after high pH stress). One hundred forty-three of these regions (94.1 %) were also identified by the MACS peak-calling algorithm, and two additional peaks, not detected by MACS were recognized by the GEM algorithm. Therefore, 145 (95.4 %) of our peaks were also identified by other standard peak-calling methods (Additional file 1: Table S1). One hundred of these regions could be specifically attributed to a single gene, whereas 52 corresponded to divergent promoters (Additional file 1: Table S1). The kinetic of Crz1 binding was very fast, with 60 % of these regions showing a maximum recruitment after 1 or 2 min (Fig. 1a). This rapid effect fits well with the reported almost instantaneous burst of cytosolic calcium [4] and the fast transition of Crz1 from cytosol to the nucleus observed upon high pH stress [17, 23]. The quantitative change in Crz1 recruitment was calculated for each intergenic region as the log2 of the ratio of the number of maximum reads at a given time and at time zero, and the result subjected to cluster analysis. As shown in Fig. 1b, five mayor clusters were obtained. All of them included gene promoters (or intergenic regions) for which there was previous evidence of calcineurin/Crz1-mediated regulation or actual binding of Crz1 was reported *in vitro* or *in vivo*.

**Fig. 2:**
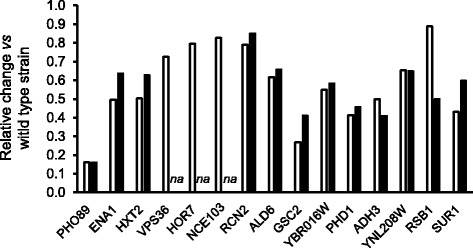
Influence of the calcineurin pathway in the transcriptional response of diverse genes recruiting Crz1 upon high pH stress. The expression levels of the indicated genes in the calcineurin-deficient *cnb1* strain (*open bars*) and the *crz1* mutant (*closed bars*) are plotted as fold-change *vs* the wild type strain. For *PHO89* the values are the mean from references [2, 4, 18]; for *ENA1*, from references [2, 4, 23, 39]; and for *HXT2 and ALD6* from references [4, 17]. For the rest of the genes, data is taken from reference [4]. *na*, not available

Seventy three of the 100 gene promoters that could be unequivocally assigned as positive for Crz1 recruitment had been previously identified as corresponding to high pH responsive genes at least in one instance [1–4, 33–35] and, for some of them, evidence for calcineurin/Crz1 mediated regulation in response to alkaline pH induction can be collected from the literature (Fig. 2). In some instances, such as *PHO89*, *ENA1, HXT2* or *ADH3*, the calcineurin/Crz1 pathway appears to be a very significant component of the response. In addition, recruitment of Crz1 to the corresponding promoters upon high pH stress was previously demonstrated or proposed [17] in nine cases (*PHO89*, *CIT2*, *HXT7*, *GSY1*, *HXT5*, *HXT2*, *ADH3*, *ALD4*, and *ALD6*). These evidences supported the robustness of our ChIP-Seq data. We also compared this 100-gene subset with the set of genes defining the calcium-mediated transcriptional response as proposed by Yoshimoto and coworkers [15]. These authors identified 934 genes induced at least 2-fold after 15 or 30 min of exposure to 0.2 M calcium chloride. We found that 43 out of the 100 promoters showing recruitment of Crz1 where induced by calcium and that in 14 cases (*PHO89*, *ENA1*, *CRH1*, *YHR097C*, *YPS1*, *YLR257W*, *TMA10*, *DIA1*, *CHS1*, *YNL208W*, *YOL014W*, *LCL1*, *SUR1*, *FLC1*) dependence on calcineurin was proposed.

Evaluation of the additional 52 intergenic regions recruiting Crz1 indicated that only in a few cases it was possible to tentatively assign the Crz1-regulated gene (e.g., on the basis of the distance of the recruitment peak to the initiating Met codon). Therefore, we decided to resort to available reported data to evaluate how likely was that a given gene could be under the control of Crz1 (Additional file 2: Table S2). We first asked how many, among the 104 possible genes, were known to be responsive to calcium stress as described by Yoshimoto and coworkers [15]. This criteria yielded 39 genes, which were added to the 100 gene list described above and included in the subsequent analyses. Among the 39 genes selected from the intergenic regions recruiting Crz1, 29 were known to be induced by high pH stress (Additional file 2: Table S2) and this subset includes several genes for which there was previous evidence of calcineurin/Crz1 mediated regulation (Fig. 2 and Additional file 2: Table S2), such as *VPS36, NCE103, GSC2, HXK1, YBR016W, YBR284W, ORM2, CMK2, or RCN2*. We found eight cases in which the regulatory role of Crz1 likely affects both genes sharing the intergenic region. These are *PRE1*/*FUI1* (possibly through two independent CDREs), *IPT1*/*SNF11*, *HSP12*/*MDJ1*, *SPS100*/*YHR140W*, *YAP1801*/*MPC2*, *NCE103*/*IDH1*, *IRC10*/*CMK2*, and *STE13*/*RCN2*. It is worth noting that the orthologues of *FUI1* and *CMK2* have been very recently shown to be positively regulated by Pzr1, the calcineurin regulated TF in fission yeast [36]. There was an additional pair of genes, *CUP2*/*PMR1*, of which *PMR1* was not identified as induced by calcium by Yoshimoto and coworkers [15]. However, there is previous evidence that this gene is induced by calcium in a Crz1-dependent fashion [11] and it has been also found to be under the control of Prz1 in *S. pombe*. In consequence, *PMR1* was included in subsequent analyses, yielding a final list of 140 genes. We also identified 10 intergenic regions for which there was no evidence in the literature of induction by high pH or by calcium stress.

### Gene Ontology analysis of genes recruiting Crz1

As described above, we generated a list of 140 genes for which recruitment of Crz1 was likely to be functionally relevant. Direct binding to and regulation by the Crz1 ortholog was reported for an identical number of genes in the rice blast fungus *Magnaporthe oryzae* [37]. This value is also quite similar to the number of genes reported by Yoshimoto and coworkers to be governed by calcineurin/Crz1 in response to calcium and salt stress. We then performed Gene Ontology analysis to identify the functional areas in which Crz1 was a relevant component. When Process Ontology was interrogated, we found an enrichment for genes related to abiotic stimulus (*p* < 0.0005) and carbohydrate metabolic process (*p* < 0.0039). This is not surprising, since Crz1 has been related in the past with processes involved in the response to stress [8]. In addition, our data confirm the early report pointing out that a calcineurin/Crz1-mediated response to high pH is involved in the induction of specific genes relevant for glucose transport and metabolism [17]. A significant subset of these proteins consists of glucose transporters (*HXT2*, *HXT7* and *HXT5*, encoding high or moderate affinity transporters) and phosphorylating enzymes (*HXK1* and *GLK1*). As shown in Fig. 1c, the kinetics of the response for these promoters was identical, with peak at 5 min upon stress (with the only exception of *HXT2*, which peaked after 1 min followed by a rapid decline). This suggests an orchestrated response aiming to improve high-moderate affinity glucose import upon high pH stress. In contrast, recruitment to promoters of other glucose transporters (*HXT4*, *HXT1* or *HXT3*) or hexose kinases (*HXK2*) was negligible.

According to Function Ontology, we observe an enrichment for genes related to sequence-specific DNA binding (*p* < 2.6e-05), mostly transcription factors either with activator (*TEC1*, *VHR1*, *PHD1*, *GIS1*, *UPC2*, *YAP6*, *CUP2*, *USV1*, *SUT2* or *CRZ1* itself) or repressor function (*XBP1*, *IXR1*, *NRG1*, and *MIG2*). This is interesting because it suggests that Crz1 might be regulating gene expression in two ways: a first response caused by direct binding to the target gene promoters and, subsequently, a secondary one, derived from the activation of diverse TFs each of them with a subset of target genes, thus likely expanding the number of genes influenced by Crz1. Such a two-step effect would not be unique as far as the high pH response is concerned, since it has been reported that certain transcriptional effects triggered by the transcription factor Rim101 are mediated indirectly through the regulation of the transcription of the Nrg1 repressor [18, 38]. It is worth noting that two of these repressors (Nrg1 and Mig2) play a role in the transcriptional response to alkaline pH stress and that there are examples of genes (*ENA1*, *PHO89*) whose promoters are regulated by Crz1 as well as by Nrg1 and Mig2 [18, 39], pointing out to an integrative response. In addition, several of these TFs, such as *TEC1*, *PHD1*, *NRG1*, *UPC2*, *MIG2*, and *SUT2* are related to filamentous growth and pseudohyphal formation. Because perturbation of Crz1/CrzA in diverse fungi leads to abnormal formation of hyphae [see [7] and references therein], our results might point to a function conserved in budding yeast. GO analysis also revealed that the list of genes recruiting Crz1 is enriched in those encoding plasma membrane proteins (*p* < 3.3e-06), which is in keeping with previous evidences indicating a role of Crz1 in ion homeostasis and nutrient utilization.

**Fig. 3:**
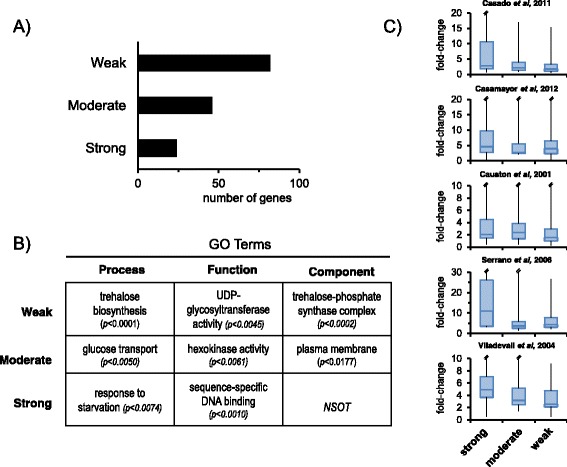
Gene ontology analysis of categorized gene promoters. **a** The 140 gene promoters selected (see text) were classified in “Strong” (>10-fold increase over the genome average reads), “Moderate” (5 to 10-fold increase), and “Weak” (2 to 5-fold change). **b** Each group of genes was subjected to Gene Ontology analysis and terms with *p*-value <0.01 were selected. Only the highest scores are presented in the Table. *NSOT*, *No Significant Ontology Term* can be assigned to this gene subset. **c** Genes were grouped according to the potency of Crz1 recruitment and the corresponding transcriptional data in response to alkaline pH stress (fold-change) were extracted from five independent datasets and presented as box plots for each dataset. Because the large dispersion of higher values and to facilitate comparison between plots the upper whisker is not shown fully in some graphs. Datasets used are: Casado et al. [34]; Casamayor et al. [35]; Causton et al. [1]; Serrano et al. [33]; and Viladevall et al. [4]. Data correspond in all cases to the response after 10 min of exposure to pH 8.0, except in reference [33], which correspond to 15 min to pH 8.2. Only genes with valid data in at least 2 datasets were used for the plots

On the basis of the number of reads accumulated at the peak region we classified the initial set of 152 promoter regions in “Strong” (more than 10-fold increase over the genome average), “Moderate” (5 to 10-fold increase), and “Weak” (2 to 5-fold change). As shown in Fig. 3a and Additional file 1: Table S1, about 50 % of the regions were classified as weak, whereas 30 % were moderate and 20 % strong. The MACS algorithm detected all regions catalogued as strong, all but one classified as moderate, and 89 % of the regions defined as weak. Interestingly, Gene Ontology analysis performed using the preselected 140-gene set showed specific profiles for each category (Fig. 3b). For instance, among weak recruiters were diverse genes involved in trehalose biosynthesis, including *UGP1* (encoding UDP-glucose pyrophosphorylase), *TPS1* (trehalose-phosphate synthase), *TPS2* (trehalose-phosphatase) and *TSL1* (encoding the large subunit of trehalose 6-phosphate synthase/phosphatase complex). It must be noted that all four genes have been consistently shown to be induced by high pH (Additional file 3: Figure S1) and the first three also by calcium (Additional file 2: Table S2). Moderate responsive genes were enriched in those encoding plasma membrane proteins, whereas strong recruiting regions corresponded in several cases to genes encoding TFs and related proteins. These results suggest a certain functional specialization regarding the ability of gene promoters to effectively recruit Crz1 and, for the first time, categorize the genes involved in trehalose metabolism as Crz1-regulated.

In addition to the calcineurin/Crz1 pathway, the transcriptional response to high pH involves the activation of many other signaling pathways [5]. We wondered whether our study could highlight the contribution of calcineurin/Crz1 signaling to the overall response. To this end, we collected transcriptional data in response to high pH stress from five different studies and the genes were grouped according to the potency of Crz1 recruitment to the corresponding promoters (Fig. 3c). Whereas no evident differences were observed between moderate and weak recruiters, those genes classified as strong recruiters in our study also display the strongest induction in most datasets, thus underscoring the relevance of the calcineurin/Crz1 pathway in the overall alkaline pH response.

### Characterization of the Crz1 binding consensus sequence

**Fig. 4:**
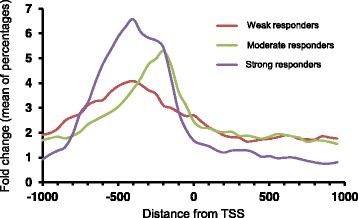
Positioning of Crz1 in promoters with strong (*blue line*), moderate (*green line*) or weak (*red line*) response. The fold-change with respect time 0 for each 50 bp bin of the region spanning 1 kb from the reported TSS were normalized as percentages of the fold-change in this region, aligned by position with respect to the TSS and the average for each 50 bp bin was calculated and plotted

We next compared the potency for recruiting the transcription factor and the relative position in the promoters of the peak of recruitment. To this end, the binding profiles for Crz1 in each group of promoters were analyzed including only those genes having a reported TSS (Fig. 4). To avoid skewness in the profiles due to the different magnitudes of fold-change, the percentage of fold-change in the region spanning 1 kb from the TSS in 50 bp bins was calculated. The profiles showed some differences: strong responder regions had the peak centered at −400 bp from the TSS, with a shoulder spanning to −200 bp. This distribution does not derive from the existence of two distinct Crz1 binding sites in these genes, but from two populations of genes with somewhat different binding positions. Weak responders also showed a peak at −400 bp. In contrast, the maximum in moderate responders was at −200 bp.

Whole genome matrix scanning for Crz1 sites revealed that putative consensus sequences could be found near the center of the peak in 118 out of 131 intergenic regions (note that in several instances the intergenic region likely regulates both flanking genes). In the rest of the cases patterns containing the conserved core sequence (C/A)GCC could also be found located near the center of the peak. In the case of *ENA1* the putative CDRE found matched with the one of higher affinity sites for Crz1 described by Mendizabal and coworkers [16]. For other genes, including those related to hexose transport (*HTX2*, *HTX5* and *HTX7*), the putative CDREs matched with those previously anticipated on theoretical grounds [17].

**Fig. 5:**
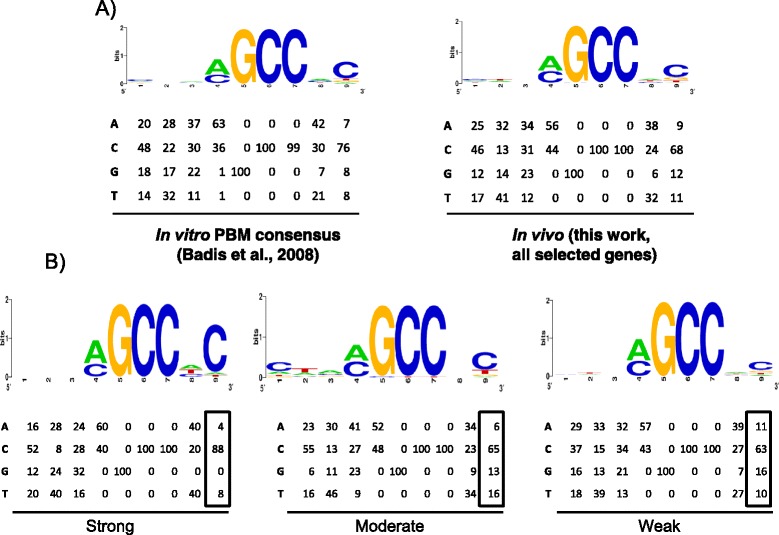
Identification of Crz1 binding motifs *in vivo*. **a** Web logo and PWM obtained for the putative CDREs of all (131) selected intergenic regions showing Crz1 binding (*right*) in comparison with the reported *in vitro* consensus [29] (*left*). **b** Web logo and PWM obtained for the putative CDREs of strong, moderate and weak promoters subsets (see Methods section)

The overall consensus sequence identified was NNN(A/C)GCCNC, with a slight prevalence of a C at the first position. The core of this sequence [(A/C)GCCNC] is reminiscent to that proposed by Yoshimoto and coworkers [15] based on *in vitro* binding selection [TG(A/C)GCCNC] or to that found as a common sequence motif [CAGCCTC] in the upstream regions of calcineurin/Crz1p-dependent genes (in the context of salt and calcium stress). However, we do not find any relevant specificity for the positions preceding the (A/C)GCC core, in contrast to that described by these authors. In fact, our *in vivo* motif selection matches very closely (Fig. 5a) with that proposed by Badis and coworkers [29] based on Protein Binding Microarray technology and indicates that, at least in this case, the *in vitro* binding data effectively correlates with *in vivo* results.

To explore the possibility that certain positions within the Crz1 putative binding sites could influence the strength of the recruitment, a position weight matrix was generated for each intensity group (Fig. 5b). The results show that in promoters with strong recruitment of Crz1 the last position in our matrix corresponds mostly with a C, with no tolerance for G, suggesting that this position, although it is not essential, probably influences the efficacy of recruitment.

### Chromatin accessibility accelerates Crz1 recruitment

**Fig. 6:**
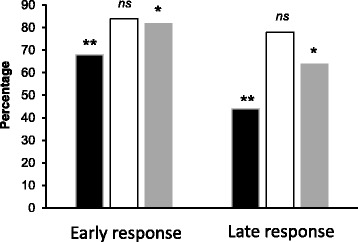
Chromatin accessibility and kinetics of Crz1 binding. Black bars correspond to the percentage of promoters where the putative CDRE was found to lie at NFRs. Empty bars and gray bars correspond to the percentage of promoters with enrichment in H3K4me3 and H3K4ac at the Crz1 recruitment peak, respectively. The data for nucleosome positioning and core histone modifications were taken from [31, 32], respectively. “Early response” includes those genes peaking at 1 or 2 min after stress, whereas “Late response” comprises those peaking at 5 or 20 min. Significance of the differences was determined using the Fisher’s exact test. *, *p* = 0.020; **, *p* = 0.006; *ns*, not significant (*p* = 0.391)

Chromatin accessibility plays a determining role in the binding of TFs. We have studied the influence of nucleosome positioning and chromatin modifications on the recruitment of Crz1. Our hypothesis was that the presence of the cognate sequence for Crz1 in DNA regions wrapped around the nucleosome core under basal conditions may difficult proper binding of the TF upon stress. We have compared the data of nucleosome positioning provided by Mavrich and coworkers [31] with the putative CDREs located in the center of the peaks obtained by ChIP-Seq (Fig. 6) and found that over 68 % of the putative CDREs in genes with early response (peaks at 1 or 2 min after stress) were located in nucleosome free regions (NFR). In contrast, in late responding genes (peaks at 5 or 20 min) only 44 % of the putative CDREs were at NFR.

Core histone post-translational modifications associated with chromatin relaxation may facilitate Crz1 recruitment. After numerous studies in the recent years regarding this topic it is generally accepted that trimethylation of H3K4 (H3K4me3) is enriched in promoter regions of actively transcribed genes, especially close to the TSS [40]. In addition, it has been described that acetylation of the same position (H3K4ac) is also enriched in *S. cerevisiae* active promoters, preferentially located upstream the H3K4me3 mark and it has been hypothesized that H3K4ac, as other acetylation marks may enhance chromatin accessibility. Alternatively, this modification may also be required for TFs to specifically promote initiation of certain genes [32]. Therefore, the regions recruiting Crz1 were compared with the data reported by Guillemette an coworkers [32] regarding the genome-wide enrichment in H3K4me3 and H3K4ac. The comparison showed that 81 % of the regions that recruited Crz1 have been found enriched in H3K4me3, while 75 % are enriched in H3K4ac. Similar results were obtained in the genome-wide analysis performed by Guillemette and co-workers, with 87 % of promoters enriched in H3K4me3 and 72 % of promoters enriched in H3K4ac. The presence of H3K4me3 was almost equally distributed among early and late response promoters, 84 and 78 %, respectively. In contrast, H3K4ac was significantly more abundant in the early response (82 %) than in the late response promoters (64 %). These results suggest that the presence of the binding site for Crz1 at NFRs and the occurrence of H3K4ac would accelerate the recruitment of this transcription factor to its target promoters by enhancing chromatin accessibility.

## Conclusions

*In vivo* binding of TF to their target genes has been investigated in the past in numerous occasions. However, in quite a few cases the universe of genes under the control of a given TF could not be characterized simply because the TF fails to bind DNA under the cell growth conditions. We show that Crz1 is very rapidly recruited to the promoters of more than 100 genes in response to high pH stress. Our work confirms and expands the relevance of Crz1 activation in the regulation of glucose-related metabolic genes. The identification of several genes encoding TF likely controlled by Crz1 might be at the basis of a signal amplification mechanism. Our results allow refining the consensus model for Crz1 binding and suggest that the presence of a C nucleotide at position +2 from the GCC core, althought not essential for binding, would enhance recruitment. Finally, localization of putative CDRE at nucleosome free regions, combined with the presence of acetylated histone H3 at Lys-4 appear to facilitate rapid access of Crz1 to the promoter.

## Additional files

Additional file 1: Table S1. Intergenic regions recruiting Crz1 in response to alkaline pH. (XLSX 39 kb) Additional file 2: Table S2. Functional analysis of divergent promoters recruiting Crz1. (XLSX 48 kb) Additional file 3: Figure S1. Alkaline pH responsiveness of genes involved in trehalose metabolism. (PPTX 64 kb)

## Abbreviations

CDRE: Calcineurin Dependent Response Element
