# Supplementary material for: Genome-wide recruitment profiling of transcription factor Crz1 in response to high pH stress
Source: BMC Genomics. 2016 Aug 20;17:662. doi: 10.1186/s12864-016-3006-6 (PMC4992276; doi:10.1186/s12864-016-3006-6)
Supplement: Additional file 3: Figure S1. — Alkaline pH responsiveness of genes involved in trehalose metabolism. (PPTX 64 kb) [file 12864_2016_3006_MOESM3_ESM.pptx]

## Slide 1
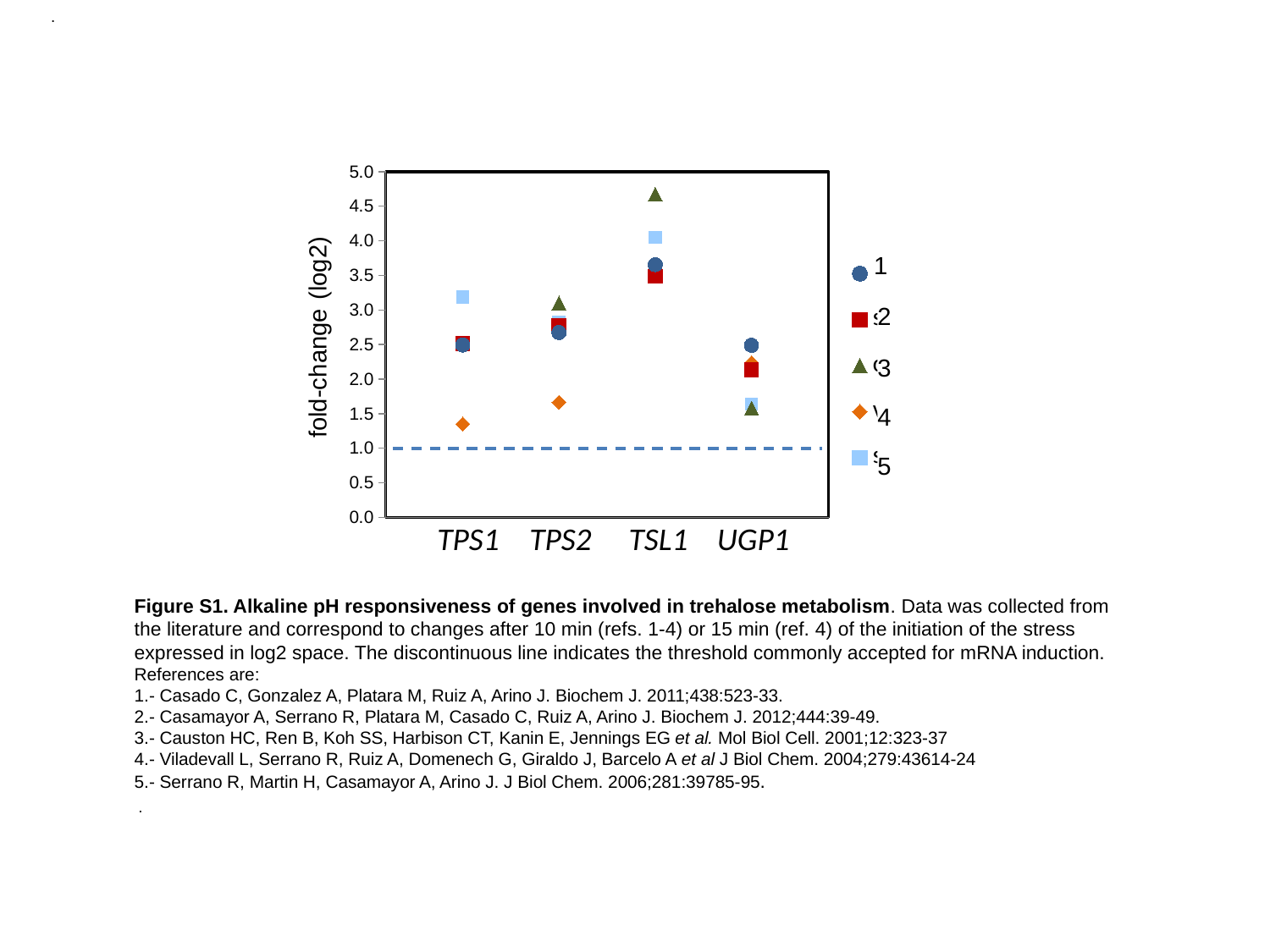

.
### Chart
| Category | | | | | |
|---|---|---|---|---|---|1
2
fold-change (log2)
3
4
5
TPS1
TPS2
TSL1
UGP1
Figure S1. Alkaline pH responsiveness of genes involved in trehalose metabolism. Data was collected from the literature and correspond to changes after 10 min (refs. 1-4) or 15 min (ref. 4) of the initiation of the stress expressed in log2 space. The discontinuous line indicates the threshold commonly accepted for mRNA induction.
References are:
1.- Casado C, Gonzalez A, Platara M, Ruiz A, Arino J. Biochem J. 2011;438:523-33.
2.- Casamayor A, Serrano R, Platara M, Casado C, Ruiz A, Arino J. Biochem J. 2012;444:39-49.
3.- Causton HC, Ren B, Koh SS, Harbison CT, Kanin E, Jennings EG et al. Mol Biol Cell. 2001;12:323-37
4.- Viladevall L, Serrano R, Ruiz A, Domenech G, Giraldo J, Barcelo A et al J Biol Chem. 2004;279:43614-24
5.- Serrano R, Martin H, Casamayor A, Arino J. J Biol Chem. 2006;281:39785-95.
.
